# Robotic-assisted minimally invasive transforaminal lumbar interbody fusion: A meta-analysis of perioperative and postoperative outcomes

**DOI:** 10.5339/qmj.2025.114

**Published:** 2025-12-11

**Authors:** Seif B. Altahtamouni, Loay A. Salman, Ahmad R. Al-Qudimat, Omar Fawaz Alnori

**Affiliations:** 1 Orthopedic Surgery Department, Hamad General Hospital, Hamad Medical Corporation, Doha, Qatar; 2 Surgical Research Section, Department of Surgery, Hamad Medical Corporation, Doha, Qatar; 3 Public Health College, QU Health, Qatar University, Doha, Qatar *Email: aalqudimat@hamad.qa

**Keywords:** Intervertebral disc, minimally invasive surgical procedures, robotics, spinal fusion

## Abstract

**Background:**

Degenerative lumbar spinal diseases are a leading cause of disability worldwide, often requiring surgical intervention when conservative management fails. Transforaminal lumbar interbody fusion (TLIF) is a commonly employed procedure to stabilize the spine and alleviate symptoms. This systematic review and meta-analysis aimed to test the safety and efficacy of robotic-assisted minimally invasive transforaminal lumbar interbody fusion (RA MIS-TLIF) in managing degenerative lumbar spinal diseases. Our primary objective was to compare the robotic approach with the conventional open or minimally invasive approach for TLIF regarding patients’ perioperative and postoperative outcomes.

**Methods:**

PubMed, Cochrane Library, Scopus, and Embase were searched from inception until October 2023. The selection criteria included only English-language articles focused on human participants aged 18 years and older with measurable outcomes. Prospective and retrospective cohort studies were included. Relevant data regarding perioperative outcomes and postoperative pain scores were then extracted. Review Manager (RevMan) 5.4 was used for statistical analysis. No restrictions were applied regarding the surgical approach compared to the robotic approach. This review was conducted based on the Preferred Reporting Items for Systematic Reviews and Meta-Analyses guidelines.

**Results:**

Twelve observational studies, including 1,385 patients, were included in our final analysis. Robotic-assisted minimally invasive lumbar interbody fusion was associated with significantly lower blood loss compared to both open (MD: −161.11 mL [95% CI, −184.89 to −137.34]) and conventional minimally invasive surgery (MD: −25.18 mL [95% CI, −64.06 to 13.70]), with an overall significant reduction (MD: −76.27 mL [95% CI, −118.65 to −33.90]). Operative time was significantly longer in robotic surgery compared to non-robotic approaches (MD: 17.61 minutes [95% CI, 4.10 to 31.11]). Hospital stay was shorter in the robotic group than in the non-robotic surgery group (MD: −0.89 days [95% CI, −1.54, −0.24]). Radiation time and dose showed no significant differences. Postoperative pain and functional outcomes, including ODI and VAS scores, showed a trend toward improvement in the robotic group but did not reach statistical significance.

**Conclusion:**

Robotic-assisted minimally invasive TLIF shows promising results in operative time and blood loss compared to open or minimally invasive TLIF.

## 1. INTRODUCTION

In recent years, lumbar degenerative diseases have been a significant concern in the medical field, which can be linked to the increased number of elderly populations worldwide.^1^ In 1982, Harms *et al* introduced open transforaminal lumbar interbody fusion (TLIF).^2^ Despite being widely used, the open technique introduced a series of challenges. Extensive paraspinal muscle detachment during a long midline incision can result in more soft tissue injury and longer hospital stays.^3^ As minimally invasive spinal surgery emerged, Seng C *et al* introduced minimally invasive transforaminal lumbar interbody fusion (MIS-TLIF) in 2002.^4^ This new technique aimed to minimize complications, blood loss, and hospitalization while achieving comparable outcomes to the open technique.^4–7^ However, MIS-TLIF showed inferiority to open TLIF in terms of operative time, learning curve, and higher risk of screw malposition and perforation, which can be attributed to the entry point’s indirect visual access.^8–12^ Moreover, MIS-TLIF was found to expose both patients and surgeons to excessive radiation due to the use of intraoperative fluoroscopy in most cases.^7,13–15^

To overcome the increased risk of screw malposition, new technologies, such as the use of an intraoperative navigation system in combination with the minimally invasive approach, have emerged.^16^ The use of navigation systems, including intraoperative computed tomography (CT) and 3D C-arm systems, enhanced the accuracy of pedicle screw placement compared to the traditional MIS-TLIF.^17–19^ Robotic guidance in spine surgery gained FDA approval in 2004 and has since gained attention among spine surgeons.^20–22^ Since then, several studies have demonstrated the advantages of robotic-assisted spine surgery, such as increased accuracy, decreased blood loss, and reduced radiation exposure to patients and surgeons.^23–25^ A meta-analysis by Fan *et al* found that the pedicle screw placement using the robotic technique was more accurate than the free-hand pedicle screw insertion.^26^

Although research in robotic-assisted surgery has advanced significantly, few studies have investigated patient outcomes in robotic-assisted minimally invasive transforaminal lumbar interbody fusion (RA MIS-TLIF). This meta-analysis aimed to test the safety and outcomes in patients with lumbar degenerative diseases who underwent RA MIS-TLIF.

## 2. MATERIALS AND METHODS

This systematic review was conducted in a stepwise manner in line with the Preferred Reporting Items for Systematic Reviews and Meta-Analyses (PRISMA) guidelines and checklist.^27^ The protocol for this review was registered in PROSPERO (International Prospective Register of Systematic Reviews) under CRD42023488139.

### 2.1 Search Strategy

A thorough search was conducted through PubMed, Cochrane Library, Scopus, and Embase. The search process took place from inception until October 17, 2023. A combination of keywords and Medical Subject Headings (MeSH) Terms with two Boolean operators was used to extract relevant articles: Robotic OR robotic-assisted OR robot-assisted OR robotic-assisted OR robot-assisted OR “Robotic Surgical Procedures” [Mesh] AND minimally invasive transforaminal lumbar interbody fusion OR MI-TLIF OR TLIF OR transforaminal lumbar interbody fusion OR minimally invasive TLIF.

### 2.2 Inclusion/Exclusion Criteria

Articles included were retrospective or prospective studies that were highly relevant to the aim of this article, with the robotic approach being the primary investigated surgery type. The criteria used for the selection included articles written in the English language only, studies conducted on humans aged 18 years and above, and studies with clearly documented and measurable outcomes. Review articles, case reports, book chapters, videos, commentaries, and conference abstracts were excluded. Studies irrelevant to our research question, including single-arm studies, were also excluded. The selection of the studies to be included in the final evaluation and review was based on relevance and the above inclusion/exclusion criteria.

### 2.3 Study Selection and Data Extraction

Study selection was done by two independent reviewers (SBA and LAS) who screened the titles and abstracts to identify relevant articles. Potentially relevant articles were then subjected to full-text screening according to the PICO (population, intervention, comparator, and outcomes) question (Table 1) and based on relevance to the inclusion criteria, and only highly relevant articles were selected for the final evaluation. Year of publication, author, study design, sample size, participants’ age and body mass index, surgery type, and follow-up periods were extracted from the selected studies. Additionally, study outcomes were extracted for statistical analysis, including operative time, estimated blood loss, length of hospital stay, radiation time, radiation dose, and postoperative pain scores.

### 2.4 Quality Assessment/Appraisal

The methodological quality of the selected studies was critically assessed using the Methodological Index for Nonrandomized Studies (MINORS).^28^ The MINORS tool is a valid instrument for assessing the quality of comparative and non-comparative nonrandomized studies. It consists of 12 domains, with the first eight being for noncomparative studies. Each domain can be awarded 0, 1, or 2, with a maximum score of 24 and 16 for comparative and noncomparative studies, respectively.

### 2.5 Statistical Analysis

Review Manager (RevMan) 5.4 was used to conduct this statistical analysis. All data were continuous and reported as mean and standard deviation. The mean difference was used with operative time, estimated blood loss, length of hospital stays, and radiation time and dose, where the standardized mean difference was used in pain scores analysis. All data were analyzed using the inverse-variance method with a 95% confidence interval (CI). Heterogeneity was tested using the chi-square test and the I^2^ index. The random-effects model was used due to the heterogeneity found between the included articles (I^2^ >50٪).

## RESULTS

### 3.1 Search Results

**Figure 1:**
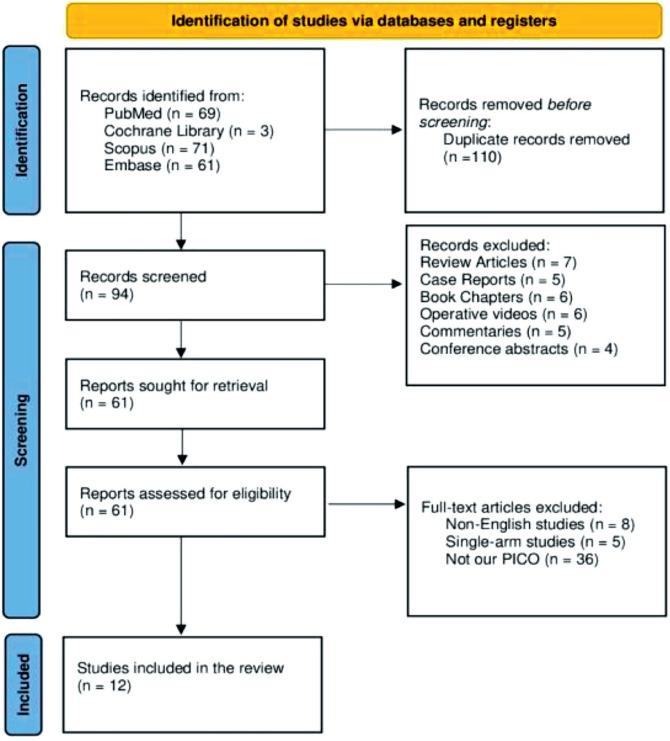
PRISMA flow diagram.

Our systematic search yielded a total of 204 articles. Of these, 110 duplicates were removed. The title and/or abstract of the remaining 94 articles were screened, and 33 were excluded. Sixty-one articles underwent full-text screening. Non-English studies, single-arm studies, and studies unrelated to our PICO question were excluded. Twelve eligible studies were included in this meta-analysis (Figure 1).

### 3.2 Study Characteristics

Twelve observational articles, including 1,385 patients, were included in this review (Table 2). Six studies were conducted in China, five in the United States, and one in Taiwan. Six articles compared robot-assisted minimally invasive lumbar interbody fusion with conventional minimally invasive interbody fusion, four compared the robotic approach with the conventional open approach, one compared robotic with minimally invasive, open, and image-guided navigation (IGN), one compared robotic to conventional and instrument-tracking minimally invasive surgery, and one compared robotic with either open or minimally invasive surgery.

### 3.3 Risk of Bias Assessment

Table 3 presents the scoring process for each study using the MINORS criteria. The studies included were scored between 17 and 22. Each study was evaluated against 12 domains and assigned a score between 0 and 2 for each domain, where 0 indicated that the domain was not reported, 1 indicated that the domain was reported but inadequately, and 2 indicated that the domain was reported and adequately. No studies were excluded from the analysis based on the MINORS criteria.

### 3.4 Perioperative Outcomes

**Figure 2:**
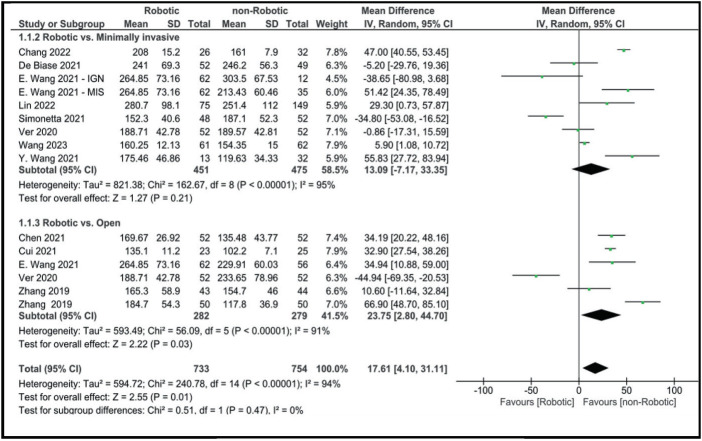
Forest plot for operative time (min).

According to our analysis, operative time was longer in the robotic approach than in the non-robotic approach (MD: 17.61 [95% CI, 4.10–31.11]; Figure 2). Operative time was also shorter in open surgery, with a mean difference of 23.75 (2.80, 44.70).

**Figure 3:**
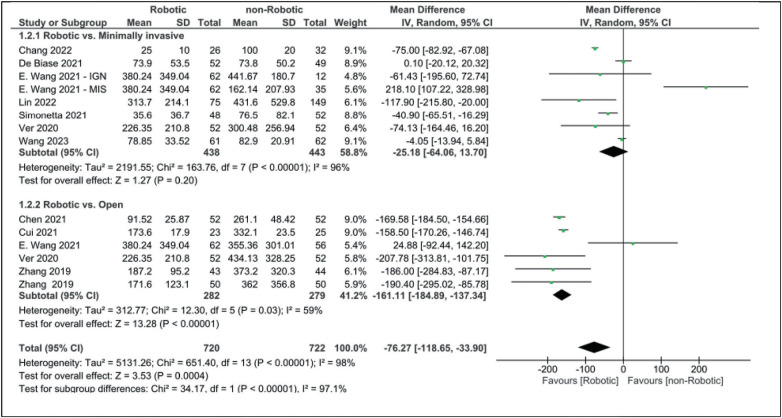
Forest plot for estimated blood loss (ml).

Compared to minimally invasive surgery, operative time was shorter than the robotic approach, although the results did not reach statistical significance (MD: 13.09 [95% CI, −7.17 to 33.35]). Blood loss was markedly higher in both open (MD: −161.11 [95% CI, −184.89 to −137.34]) and minimally invasive surgery (MD: −25.18 [95% CI, −64.06 to 13.70]) when compared to robotic-assisted surgery (Figure 3).

**Figure 4:**
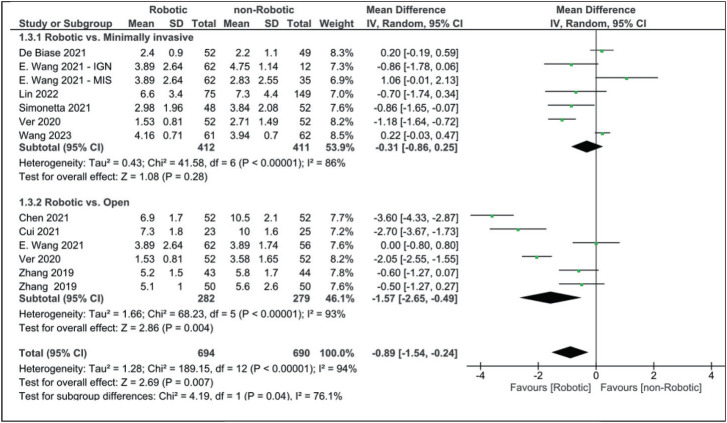
Forest plot for length of hospital stay (days).

When comparing robotic and non-robotic surgeries, blood loss is less in robotic surgery with marked statistical significance (MD: −76.27 [95% CI, −118.65 to −33.90]). Patients who have undergone robotic-assisted surgery spent less time in the hospital than those who have undergone open (MD: −1.57 [95% CI, −2.65 to −0.49]) or minimally invasive surgery (MD: −0.31 [95% CI, −0.86 to 0.25]). The mean difference between robotic and non-robotic surgery in terms of hospital stay length was −0.89 (−1.54, −0.24), which appears to be reaching statistical significance (Figure 4).

**Figure 5:**
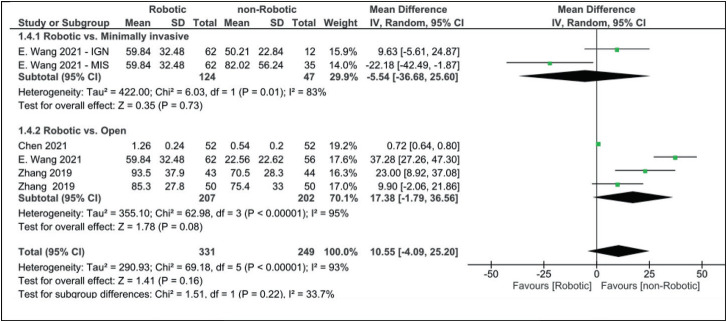
Forest plot for radiation time (min).

Robotic-assisted surgery radiation time appeared to be longer than in minimally invasive surgery but shorter than the open approach, with a mean difference of −5.54 (−36.68, 25.60) and 17.38 (−1.79, 36.56), respectively (Figure 5).

**Figure 6:**
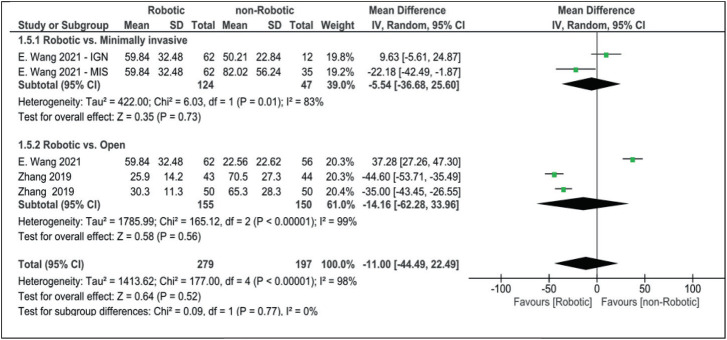
Forest plot for radiation dose (μSv).

Our analysis for radiation time did not reach statistical significance (robotic vs. non-robotic MD: 10.55 [95% CI, −4.09 to 25.20]). Only three articles examined the difference in the administered radiation dose between the robotic and non-robotic approaches. The radiation dose was slightly lower in the robotic group (MD: −11.00 [95% CI, −44.49 to 22.49]), although it did not reach statistical significance (Figure 6).

### 3.5 Postoperative Outcomes

**Figure 7:**
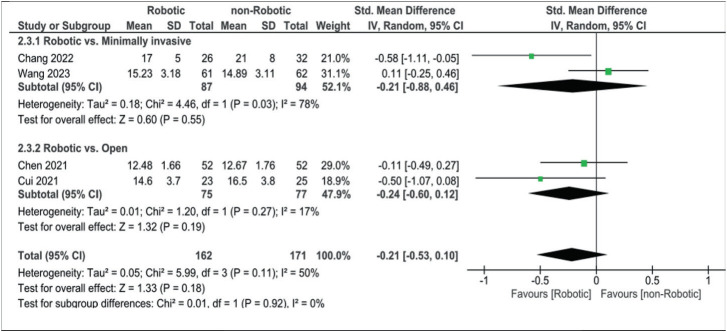
Forest plot for Oswestry Disability Index.

Four articles commented on postoperative outcomes using the Oswestry Disability Index (ODI), visual analog scale (VAS) for low back pain, and VAS for leg pain. For ODI, our analysis revealed a standardized mean difference of −0.21 (−0.53, 0.10) when comparing robotic-assisted surgery with all non-robotic approaches, −0.24 (−0.60, 0.12) when comparing it to open alone, and −0.21 (−0.88, 0.46) when comparing it to minimally invasive surgery alone (Figure 7).

**Figure 8:**
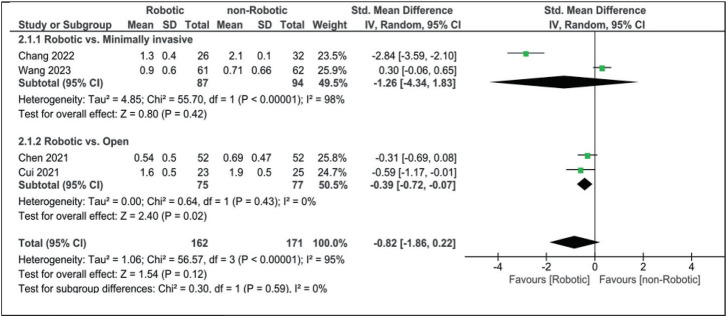
Forest plot for Visual Analogue Scale for low back pain.

VAS for low back pain was slightly better in the robotic group than in both open and minimally invasive surgery groups (robotic vs. open SMD: −0.39 [95% CI, −0.72 to −0.07] and robotic vs. minimally invasive SMD: −1.26 [95% CI, −4.34 to 1.83]). When comparing robotics with non-robotic in general, VAS was still better among the robotic group (SMD: −0.82 [95% CI, −1.86 to 0.22]), although it did not reach statistical significance (Figure 8).

**Figure 9:**
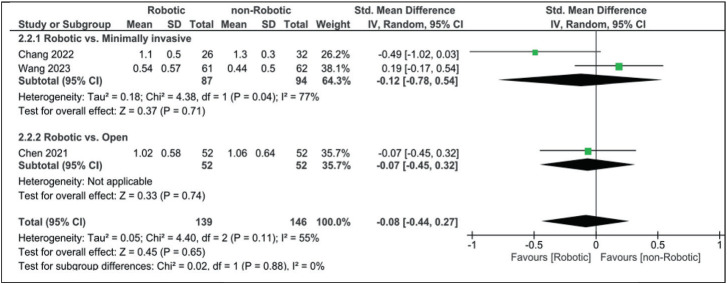
Forest plot for Visual Analogue Scale for leg pain.

As for VAS for leg pain, results did not reach statistical significance, with slight differences between both groups (robotic vs. open SMD: −0.07 [−0.45, 0.32], robotic vs. minimally invasive SMD: −0.12 [−0.78, 0.54], and robotic vs. non-robotic SMD: −0.08 [−0.44, 0.27]; Figure 9).

## 4. DISCUSSION

We conducted a systematic review and meta-analysis of 12 observational studies involving 1385 patients to gain insight into the use of robotic-assisted surgery in patients suffering from lumbar degenerative diseases in terms of patients’ perioperative and postoperative outcomes. We found that robotic-assisted transforaminal lumbar interbody fusion (RA MIS-TLIF) shows promising results in terms of operative time, intraoperative blood loss, length of hospitalization, and radiation time and dose, with comparable postoperative outcomes to both open and minimally invasive TLIF.

Lumbar interbody fusion can be carried out using different approaches. Transforaminal interbody fusion is among the most common approaches, including open or minimally invasive surgery. Several studies have shown the superiority of MIS-TLIF over open TLIF. For example, hardware placement in MIS-TLIF is found to be more accurate than in open TLIF, which in turn can minimize soft tissue damage, perform bilateral decompression through a unilateral approach, and maintain stabilization of the posterior spinal segment.^41^ By minimizing damage and being more accurate with hardware placement and dissection, MIS-TLIF can help patients undergo less collateral trauma during surgery, resulting in improved surgical outcomes.^35^

Recently, the use of robotic systems with MIS-TLIF in treating lumbar degenerative diseases has become widely studied in the literature.^32,39,42–45^ This new approach has been adopted to assess surgeons in pedicle screw placement and make spine surgeries more efficient and safer.^46,47^ Another reason for the emergence of robotic-assisted surgery is the presumed ability to reduce radiation exposure for patients and surgeons, which is a major concern with minimally invasive surgeries.^48^ RA MIS-TLIF allows surgeons to be more accurate and precise,^42^ and without the need to expose anatomical markers to determine an entry point, direction, and length of pedicle screws, which decreases muscle damage.^31^ These factors can reflect early ambulation after surgery, which lowers the rate of complications related to prolonged bed rest.^31^ Furthermore, due to its high precision, the revision rate following RA MIS-TLIF can be lower than other surgical approaches.^49^

Although some studies reported no difference between the RA technique and freehand technique in terms of operative time,^50^ most studies found an increase in operative time in the RA technique.^51–53^ A systematic review by Ghasem *et al* found increased operative time during surgeries that used robotic guidance.^54^ An increase in operative time in the RA MIT-TLIF surgery compared to either open or minimally invasive surgery in previous articles aligns with our findings (MD: 17.61 [95% CI, 4.10–31.11]). This increase in operative time can be explained by several reasons, including the need for more intraoperative preparation and usage of the robot system and the initial learning curve for the procedure.^29^ On the other hand, estimated blood loss along with the length of hospital stay was found to be significantly lower in the robotic group in our analysis (MD: −76.27 [95% CI, −118.65 to −33.90] and MD: 0.89 [−1.54 to −0.24], respectively). This can be explained by the lack of dissection while using the robotic arm, which allows the surgeon to only incise the skin and fascia, limiting further tissue damage.^55^ Previous studies have also demonstrated that RA MIS-TLIF can induce fast recovery, lowering hospital stays and costs.^56,57^ In our analysis, VAS scores for leg and lower back pain, in addition to ODI scores after at least 12 months, were comparable between robotic and non-robotic approaches. However, some studies show that patients in robotic groups showed better scores one month after surgery.^30^

Despite the high hopes of robotic-assisted spine surgery, its limitations and obstacles must be recognized. One of the leading concerns is the economic aspect. Robotic systems are expensive, require ongoing maintenance, and are operated by surgical teams needing special training. These costs may impede accessibility and uptake, especially in low-resource settings or smaller sub-sector facilities.^58,59^ Longer operative time might also be associated with higher costs related to health care resources and lower operating room efficiency, especially at the start of implementation.^54^ Additionally, the learning curve for robotic-assisted surgery remains a significant hurdle. Although several reports have suggested that surgeons’ learning curve for robotic-assisted procedures may not be too steep,^31,32^ up to 20 to 40 cases have been reported to be necessary to ensure enough procedural success.^59–64^ Such variability could affect surgery’s performance in the early learning curve. Of note, even though robotic systems are generally thought to decrease the radiation the surgical team is exposed to, we could not find any statistical difference in our meta-analysis in radiation time or dose between the robotic approach and traditional devices. Hence, the assumed advantage in this area may not be universal among all clinical setups or robotic systems.^25,30,39,50^

### 4.1 Limitations

To our knowledge, this is the first meta-analysis testing patients’ outcomes after robotic-assisted surgeries for lumbar degenerative diseases. Our study has limitations, including the retrospective and prospective design of the included studies, which inherently introduce a degree of bias to our analysis. The small sample size across all studies also limits the accuracy of our results. Another important limitation is the heterogeneity in the results of the studied variables, which lowered the degree of evidence. Furthermore, the introduction of subgroups did not reduce the heterogeneity.

## 5. CONCLUSION

This meta-analysis shows promising results for the use of robotic-assisted surgery in the management of lumbar degenerative diseases in terms of operative time, estimated blood loss, and length of hospital stay, with slightly better results regarding radiation time and dose and comparable postoperative outcomes to both minimally invasive and open transforaminal lumbar interbody fusion.

## AVAILABILITY OF DATA, CODE, AND MATERIALS

Available upon request.

## ETHICS APPROVAL

No ethical approval is required.

## AUTHOR CONTRIBUTIONS

All authors contributed to the study’s conception and design. Material preparation, literature review, data collection, and quality assessment were performed by SBA and LAS. Statistical analysis was performed by SBA. The first draft of the manuscript was written by SBA, and all authors commented on previous versions of the manuscript. All authors read and approved of the final manuscript.

## CONFLICT OF INTEREST

The authors have no relevant financial or non-financial interests to disclose. The authors declare that no funds, grants, or other support were received during the preparation of this manuscript.

## Figures and Tables

**Table 1.** PICO question elements.

Study	Population	Intervention	Comparator	Outcome of interest
Chang 2022^29^	Single-level lumbar spinal stenosis, spondylolisthesis	RA MIS-TLIF	MIS-TLIF	Operative time, EBL, VAS for low back pain, VAS for Leg pain, ODI
Chen 2021^30^	Single-level lumbar disc herniation, spinal stenosis, and spondylolisthesis	RA MIS-TLIF	Open TLIF	Operative time, EBL, LOS, Radiation time, VAS for low back pain, VAS for leg pain, ODI
Cui 2021^31^	Single level lumbar spondylolisthesis	RA MIS-TLIF	Open TLIF	Operative time, EBL, LOS, VAS for low back pain, ODI
De Biase 2021^32^	Single and multilevel lumbar degenerative disease	RA MIS-TLIF	MIS-TLIF	Operative time, EBL, LOS, Radiation time
E. Wang 2021^33^	Single and multilevel lumbar degenerative disease	RA MIS-TLIF	IGN MIS-TLIF, Open TLIF, MIS-TLIF	Operative time, EBL, LOS, Radiation time, Radiation dose
Lin 2022^34^	Multilevel lumbar degenerative disease	RA MIS-TLIF	MIS-TLIF	Operative time, EBL, LOS
Simonetta 2021^35^	single and multilevel lumbar spondylolisthesis, disc herniation, and stenosis	RA MIS-TLIF	MIS-TLIF	Operative time, EBL, LOS
Ver 2020^36^	Single and multilevel lumbar spondylolisthesis, stenosis, and mechanical disc collapse	RA MIS-TLIF	MIS-TLIF, Open TLIF	Operative time, EBL, LOS
Wang 2023^37^	Single and multilevel lumbar degenerative disease	RA MIS-TLIF	MIS-TLIF	Operative time, EBL, LOS, VAS for back pain, VAS for leg pain, ODI
Y. Wang 2021^38^	Single and multilevel lumbar degenerative disease	RA MIS-TLIF	IT MIS-TLIF, MIS-TLIF	Operative time
Zhang 2019^39^	Single-level lumbar degenerative disease	RA MIS-TLIF	Open TLIF	Operative time, EBL, LOS, Radiation time, Radiation dose
Zhang 2019^40^	Single-level lumbar degenerative disease	RA MIS-TLIF	Open TLIF	Operative time, EBL, LOS, Radiation time, Radiation dose

Abbreviations: RA, Robotic-assisted; IGN, Image-guided navigation; IT, Instrument tracking; MIS-TLIF, Minimally invasive transforaminal lumbar interbody fusion; EBL, Estimated blood loss; LOS, Length of hospital stay; VAS, Visual analogue scale; ODI, Oswestry disability index.

**Table 2.** Characteristics of included articles.

Study	Design	Intervention	Sample size	Age (mean ± SD), years	BMI (mean ± SD)	Follow-up (mean ± SD), months
Chang 2022^29^	Prospective	RA MIS-TLIF vs	26	57.2 ± 13.5	NA	17.1 ± 8.5
		MIS-TLIF	32	56.1 ± 12.1	NA	18.6 ± 7.6
Chen 2021^30^	Retrospective	RA MIS-TLIF vs	52	57.98 ± 12.63	24.86 ± 3.82	15.81 ± 3.33
		Open TLIF	52	58.08 ± 9.87	23.71 ± 2.68	16.62 ± 3.38
Cui 2021^31^	Retrospective	RA MIS-TLIF vs	23	51.3 ± 9.8	NA	NA
		Open TLIF	25	54.1 ± 10.2	NA	NA
De Biase 2021^32^	Retrospective	RA MIS-TLIF vs	52	56 ± 11.7	28.8 ± 4.8	6.1 ± 3.8
		MIS-TLIF	49	58.7 ± 10.5	30.3 ± 4.6	10.6 ± 8.2
E. Wang 2021^33^	Retrospective	RA MIS-TLIF vs	62	61.74 + 11.78	28.79 + 6.97	NA
		IGN MIS-TLIF vs	12	63.42 + 8.56	27.1 + 3.36	NA
		Open TLIF vs	56	60.63 + 13.9	29.82 + 6.67	NA
		MIS-TLIF	35	50.63 + 12.48	30.78 + 7.1	NA
Lin 2022^34^	Retrospective	RA MIS-TLIF vs	75	65.38 ± 10.02	25.8 ± 3.9	NA
		MIS-TLIF	149	62.7 ± 12.61	26.1 ± 4.0	NA
Simonetta 2021^35^	Retrospective	RA MIS-TLIF vs	48	58.2 ± 13.1	33.0 ± 6.8	NA
		MIS-TLIF	52	56.3 ± 13.2	32.5 ± 5.0	NA
Ver 2020^36^	Retrospective	RA MIS-TLIF vs	52	54.46 ± 12.95	30.60 ± 6.78	NA
		MIS-TLIF vs	52	54.44 ± 12.92	31.54 ± 6.36	NA
		Open TLIF	52	53.08 ± 12.21	32.15 ± 8.26	NA
Wang 2023^37^	Prospective	RA MIS-TLIF vs	61	57.46 ± 8.68	24.46 ± 3.45	NA
		MIS-TLIF	62	57.69 ± 9.15	25.23 ± 3.62	NA
Y. Wang 2021^38^	Retrospective	RA MIS-TLIF vs	13	68.50	31.78	NA
		IT MIS-TLIF vs	32	66.76	29.93	NA
		MIS-TLIF	74	67.49	32.04	NA
Zhang 2019^39^	Prospective	RA MIS-TLIF vs	43	56.7 ± 12.5	26.4 ± 3.9	NA
		Open TLIF	44	60.2 ± 10.9	25.2 ± 2.8	NA
Zhang 2019^40^	Prospective	RA MIS-TLIF vs	50	54.6 ± 11.1	25.6 ± 3.9	NA
		Open TLIF	50	55.6 ± 12.8	25.3 ± 3.1	NA

Abbreviations: NS, Not stated; RA, Robotic-assisted; IGN, Image-guided navigation; IT, Instrument tracking; MIS-TLIF, Minimally invasive transforaminal lumbar interbody fusion.

**Table 3.** MINORS tool for risk of bias assessment.

Study	Clearly stated aim	Inclusion of consecutive patients	Prospective of data collection	Endpoints appropriate to the study aim	Unbiased assessment of the study endpoint	Follow-up period appropriate to the study aim	<5% lost to follow-up	Prospective calculation of study size	Adequate control group	Contemporary groups	Baseline equivalence of groups	Adequate statistical analyses	Total
Chang 2022^29^	2	2	2	2	1	2	2	2	1	2	1	2	21/24
Chen 2021^30^	2	2	2	2	1	2	0	2	2	1	2	2	20/24
Cui 2021^31^	2	2	2	2	2	2	2	2	2	2	1	2	22/24
De Biase 2021^32^	2	2	1	2	0	1	2	2	2	1	2	2	19/24
E. Wang 2021^33^	2	2	2	2	0	0	2	2	1	2	1	2	18/24
Lin 2022^34^	2	2	2	2	0	1	2	2	1	2	1	2	19/24
Simonetta 2021^35^	2	1	2	2	1	0	2	2	2	1	1	2	18/24
Ver 2020^36^	2	2	2	2	1	0	2	2	2	0	2	2	19/24
Wang 2023^37^	2	2	2	2	0	2	2	2	2	2	2	2	22/24
Y. Wang 2021^38^	2	2	2	2	1	0	2	1	1	1	1	2	17/24
Zhang 2019^39^	2	2	1	2	1	0	2	2	2	1	1	2	18/24
Zhang 2019^40^	2	2	2	2	2	0	2	2	2	2	1	2	21/24
